# Supplementary material for: Impact of type of dialyzable beta-blockers on subsequent risk of mortality in patients receiving dialysis: A systematic review and meta-analysis
Source: PLoS One. 2022 Dec 30;17(12):e0279680. doi: 10.1371/journal.pone.0279680 (PMC9803304; doi:10.1371/journal.pone.0279680)
Supplement: S1 Text — (DOCX) [file pone.0279680.s002.docx]

**Search strategies for PubMed/MEDLINE, EMBASE and** [**Cochrane**](http://www.cochranelibrary.com/) **library**

**Appendix.**

Search strategies for the different databases ran on February 28, 2022.

**PubMed/MEDLINE Search Query**

("adrenergic beta antagonists"[Pharmacological Action] OR "adrenergic beta antagonists"[MeSH Terms] OR ("adrenergic"[All Fields] AND "beta antagonists"[All Fields]) OR "adrenergic beta antagonists"[All Fields] OR ("adrenergic"[All Fields] AND "beta"[All Fields] AND "antagonists"[All Fields]) OR "adrenergic beta antagonists"[All Fields]) AND ("renal dialysis"[MeSH Terms] OR "dialysis"[MeSH Terms])

**EMBASE**

('dialysis'/exp OR dialysis) AND ('adrenergic beta receptors' OR 'beta-blockers' OR 'beta-blocker'/exp OR 'beta-blocker')AND('clinical article'/de OR 'clinical study'/de OR 'clinical trial'/de OR 'cohort analysis'/de OR 'controlled clinical trial'/de OR 'controlled study'/de OR 'observational study'/de OR 'outcomes research'/de OR 'prospective study'/de OR 'randomized controlled trial'/de OR 'randomized controlled trial topic'/de OR 'retrospective study'/de)

**Cochrane Library**

ID Search Hits

#1 (Adrenergic beta-Antagonists):ti,ab,kw (Word variations have been searched)

#2 dialysis

#3 #1 and #2
