# Supplementary material for: Impact of type of dialyzable beta-blockers on subsequent risk of mortality in patients receiving dialysis: A systematic review and meta-analysis
Source: PLoS One. 2022 Dec 30;17(12):e0279680. doi: 10.1371/journal.pone.0279680 (PMC9803304; doi:10.1371/journal.pone.0279680)
Supplement: S1 Table — (DOCX) [file pone.0279680.s003.docx]

**Quality assessment of the included studies**

**S1 Table. Newcastle-Ottawa Scale Quality Assessment of included studies**

|  | **Selection** | | | | **Comparability** | | **Exposure** | | | | |  |
| --- | --- | --- | --- | --- | --- | --- | --- | --- | --- | --- | --- | --- |
| First author / Year | **Representativeness of the exposed cohort** | **Selection of the non exposed cohort** | **Ascertainment of exposure** | **Demonstration that outcome of interest was not present at start of study** | **Comparability of cohorts on the basis of the design or analysis** | | **Assessment of outcome** | **Was follow-up long enough for outcomes to occur** | | | **Adequacy of follow up of cohorts** | **Total** |
| Matthew Weir / 2015 | * | * | * | * | | ** | * | | - | * | | 8 |
| Theresa Shireman / 2016 | * | * | * | * | | ** | * | | * | * | | 9 |
| Magdalene Assimon/  2018 | * | * | * | * | | ** | * | | * | * | | 9 |
| Ping-Hsun Wu / 2020 | * | * | * | * | | ** | * | | * | * | | 9 |
