# Supplementary material for: Impact of type of dialyzable beta-blockers on subsequent risk of mortality in patients receiving dialysis: A systematic review and meta-analysis
Source: PLoS One. 2022 Dec 30;17(12):e0279680. doi: 10.1371/journal.pone.0279680 (PMC9803304; doi:10.1371/journal.pone.0279680)
Supplement: S2 Table — (DOCX) [file pone.0279680.s004.docx]

**S2 Table. Sensitivity analyses for all-cause mortality**

| **Sensitivity analysis** | **No. of studies** | **Pooled OR (95%CI)** | **P value** | **I^2^** |
| --- | --- | --- | --- | --- |
| Exclusion of a study including peritoneal dialysis patients | | | | |
| Before exclusion | 4 | 0.94 (0.77-1.15) | 0.55 | 92.81% |
| After exclusion | 3 | 0.92 (0.72-1.18) | 0.52 | 94.32% |
| Exclusion of a study categorized bisoprolol as LDBBs | | | | |
| Before exclusion | 4 | 0.94 (0.77-1.15) | 0.55 | 92.81% |
| After exclusion | 3 | 0.85 (0.71-1.02) | 0.09 | 91.72% |

Abbreviations: LDBBs, low dialyzable beta-blockers
