# Supplementary material for: Impact of type of dialyzable beta-blockers on subsequent risk of mortality in patients receiving dialysis: A systematic review and meta-analysis
Source: PLoS One. 2022 Dec 30;17(12):e0279680. doi: 10.1371/journal.pone.0279680 (PMC9803304; doi:10.1371/journal.pone.0279680)
Supplement: S3 Table — (DOCX) [file pone.0279680.s005.docx]

**S3 Table. Meta-regression analysis**

| **Moderators** | **Coefficient** | **SE** | **Z value** | **P value** | **95% CI** |
| --- | --- | --- | --- | --- | --- |
| **All-cause mortality** | | | | | |
| CAD | 0.0124 | 0.0075 | 1.65 | 0.10 | -0.0023-0.0272 |
| HF | 0.0242 | 0.0189 | 1.28 | 0.20 | -0.0129-0.0612 |

Abbreviation: CI, confidence interval; CAD, coronary artery disease; HF, heart failure; SE, standard error
