# Supplementary figures and images for: Impact of type of dialyzable beta-blockers on subsequent risk of mortality in patients receiving dialysis: A systematic review and meta-analysis
Source: PLoS One. 2022 Dec 30;17(12):e0279680. doi: 10.1371/journal.pone.0279680 (PMC9803304; doi:10.1371/journal.pone.0279680)

**S1 Figure. The GRADE results**


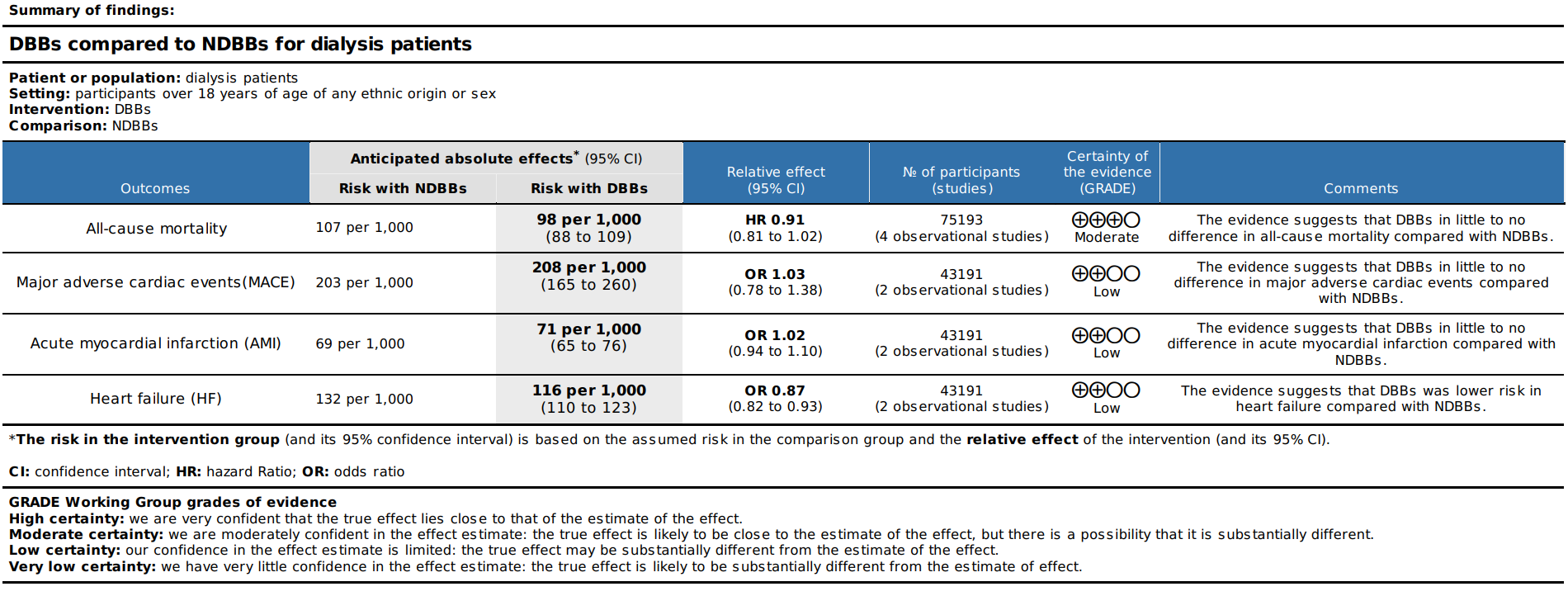

Supplement: S1 Fig — (DOCX) [file pone.0279680.s006.docx]
