# Supplementary material for: Impact of type of dialyzable beta-blockers on subsequent risk of mortality in patients receiving dialysis: A systematic review and meta-analysis
Source: PLoS One. 2022 Dec 30;17(12):e0279680. doi: 10.1371/journal.pone.0279680 (PMC9803304; doi:10.1371/journal.pone.0279680)
Supplement: Attachment — Submitted filename: response to reviewers.docx [file pone.0279680.s007.docx]

12/05/2022

Emily Chenette

Editor in Chief

*PLOS ONE*

RE:

Manuscript ID: PONE-D-22-24265

Manuscript title: **Impact of type of dialyzable beta-blockers on subsequent risk of mortality in patients receiving dialysis: A systematic review and meta-analysis**

Dear Editor,

On behalf of all the co-authors, I would like to express my sincere gratitude to the reviewers and the staff at the Editorial office for your time and efforts devoted to our manuscript. Below please find a copy of our responses and revised manuscript to the constructive comments from the reviewers and Editorial office regarding our manuscript ID: PONE-D-22-24265; with manuscript title: **Impact of type of dialyzable beta-blockers on subsequent risk of mortality in patients receiving dialysis: A systematic review and meta-analysis**. It’s ready to be re-submitted to the prestigious **PLOS ONE**. We would be like to emphasize that: We had enrolled four studies. The pooled results demonstrated that dialysis patients had no significant risk of mortality, MACE between taking dialyzable high beta-blockers and low dialyzable beta-blockers. On the other hand, we had modified terms of high dialysable beta-blockers (HDBBs)/ low dialysable beta-blockers (LDBBs) to dialyzable beta-blockers (DBBs)/ non-dialyzable beta-blockers (NDBBs) in the modified version of manuscript after English editing. We believe that with the significant modifications made to be compatible with the high standards and great recommendations set by the reviewers, this article is now more valuable, and ready for the readers of **PLOS ONE**.

Sincerely yours,

Jui-Yi Chen, MD

Division of Nephrology, Department of Internal Medicine, Chi Mei Medical Center,

Tainan, Taiwan

No. 901, Zhonghua Rd., Yongkang Dist., Tainan City, Taiwan

E-mail: kwuilus0101@gmail.com

To reviewer 1

#1. The English needs to be improved throughout the paper prior to being suitable for publication. Response:

Thanks for your suggestion. Our manuscript had been edited by experts in English.

#2. The study compares high vs. low dialyzable beta blockers. It would be helpful to inform the reader what threshold is used to determine high vs. low dialyzability.

Response:

Thank you for your comment. There was no definite cut-off value for determination of high dialyzable and low dialyzable beta blockers[1-4]. We had modified terms of high dialysable beta-blockers (HDBBs)/ low dialysable beta-blockers (LDBBs) to dialyzable beta-blockers (DBBs)/ non-dialyzable beta-blockers (NDBBs) in the modified version of manuscript. Moreover, the dialyzable thresholds of the beta-blockers were not mentioned in all included studies. According to Tieu’s study[1], only 10% of medication had reported definitive information about the threshold for drugs’ dialyzability. The classification of cardio-selective BBs and non-cardio-selective BBs in Shireman and Assimon’s studies[5, 6], it is also the categories by the dialyzability according to several studies about pharmacokinetics of beta-blockers[1, 2]. (DBBs, Cardio-selective BBs: Atenolol, metoprolol, nadolol; NDBB, Non-Cardio-selective: carvedilol, labetalol, and propranolol). Additionally, the classification of DBBs and NDBBs was based on studies of pharmacokinetics of beta-blockers[7-12] in Weir’s and Wu’s study[13, 14]. We had added the description for the detailed information in the introduction and result as following, Line 66” *However, there was no definite threshold for differentiating between DBBs and NDBBs.*”, Line 169*” The classification of DBBs and NDBBs was based on the pharmacokinetics of beta-blockers (17-22) in two of the included studies (10, 12), while the classification of the other two studies was based on the cardio-selectivity of beta-blockers (13, 23). Previous studies have shown that cardio-selective and non-cardio-selective beta-blockers are pharmacologically equivalent to DBBs and NDBBs, respectively (11, 24).”*

#3. I may have missed this but how was MACE defined? I assume acute MI and heart failure were included in this (though they are also listed as separate outcomes? Were any other cardiovascular outcomes included in MACE?

Response:

Thank you for your comment. Several MACE definitions had been elucidated [15] including 3-point MACE, 4-point MACE and 5-point MACE. The classical 3-point MACE is composed of nonfatal stroke, nonfatal myocardial infarction, and cardiovascular death. The 4-point MACE is defined as classical 3-point MACE, with other events, like hospitalization for unstable angina or revascularization procedures. Finally, the definition of 5-point MACE is the result of 4-point MACE, and heart failure.[15] However, the MACE in the included studies were a little bit different, and it had been expressed at the response Table 1. Separately.

Response Table 1.

|  | MACE definition |
| --- | --- |
| Weir (2015) | cardiovascular death, myocardial infarction, heart failure |
| Wu (2020) | acute coronary syndrome, ischemic stroke and heart failure |

Abbreviations: MACE, major adverse cardiovascular event

We had added the description for the detailed information in the result, line 117, as following, “*The definition of MACE was based on that of each included study.*” And added on” *There was a slight variation in the definition of overall MACE between the two studies; while it involved cardiovascular death, myocardial infarction, and heart failure in one of the studies (12), the other referred it to acute coronary syndrome, ischemic stroke, and heart failure (10).*” in line 231.

#4. One of the included studies included peritoneal dialysis patients. I find it questionable of whether to include these patients given the inherent differences in dialysis modalities. For instance, given the much slower/prolonged clearance with peritoneal dialysis, one would think the contribution of intradialytic hypotension from beta-blocker clearance would be much less significant in this population.

Response:

Thanks for your comment. Theoretically, the blood concentration of a drug would be steadier in peritoneal dialysis patient than in the hemodialysis patients due to the much slower/prolonged clearance of peritoneal dialysis. However, there are less studies discussing about the relation between drug dialyzability and peritoneal dialysis. According to Hirata’s study,[16] drug clearance for a given duration is markedly lower during CAPD than hemodialysis. Nevertheless, CAPD is a continuous procedure. Therefore, weekly clearance is not markedly different between CAPD patients and hemodialysis patients. On the other hand, we had provided sensitivity analysis which exclude the study composed of peritoneal dialysis population (S2 Table. Sensitivity analysis), and displayed no significant difference after sensitivity analysis.

#5. I am confused by the heart failure results. The abstract states that "HDBBs were associated with significant reduction of risk in HF compared with individuals with LDBBs". However, when I look at Fig 2D which is showing heart failure outcomes seems to show that the results favor LDBBs which seems contrary to the prior statement. Further, this conclusion is drawn from only two studies, one (Wu) which contributes 97% of the relative weight while the other (Weir) is questionable as per the authors as bisoprolol may have been included in the wrong HDBB/LDBB category in that study. This draws into question any conclusions that can be drawn from this analysis.

Response:

Thanks for your comment. We had modified the Fig 2. Also, we had added on reviewer’s comments at discussion in line 370 as following,” *However, this conclusion was drawn from only two studies, one (10) of which contributed to 97% of the relative weight while the finding of the other (12) was questionable because the authors classified bisoprolol into the DBB category.*”

To reviewer 2

#1. Abstract. Sentence in the results from "The risk of MACE to............]." appeared of no clear meaning and truncated

Response:

Thanks for your comment. We had revised as the following (line 30)” *Analysis of four observational studies including 75,193 individuals undergoing dialysis in hospital and community settings after a follow-up from 180 days to six years showed an overall all-cause mortality rate of 11.56% (DBBs and NDBBs: 12.32% and 10.7%, respectively) without significant differences in risks of mortality between the two groups [random effect, aHR 0.91 (95% CI, 0.81–1.02), p=0.11], overall MACE [OR 1.03 (95% CI, 0.78–1.38), p=0.82], and AMI [OR 1.02 (95% CI, 0.94–1.1), p=0.66].*”

#2. Abstract. It is not clear if the present results were in hospital or at which time of follow up

Response:

Thanks for your comment. We had added on detailed description at line 30 as following” *Analysis of four observational studies including 75,193 individuals undergoing dialysis in hospital and community settings after a follow-up from 180 days to six years*”

#3. Methods. It is not clear if results derived from multivariable adjustement or not

Response:

Thanks for your comment. We had revised the description in method (line 140) as” *The odds ratio (OR) and 95 % confidence interval (CI) were extracted from the included studies. In addition, the hazard ratio (HR) of mortality was also analyzed. For studies that provided information on HR, the data were used for analysis.*” We not only expressed the pooled result with aHR (Fig 2A) [aHR: 0.91; 95% CI, 0.81–1.02, p = 0.11], but also demonstrated a consistent result for mortality with OR (S6 appendix).

| S6 appendix. Forest plot for mortality (Odds ratio) | Fig 2A. Forest plot for mortality (Hazard ratio) |
| --- | --- |
|  |  |

#3. Methods. Meta-regression for anamnesis of CAD, of HF and of EF (if available) should be added

Response:

Thanks for your comment. We had added meta-regression for anamnesis of CAD and of HF. (S3 Table 3) However, there was no EF data available. We had added meta-regression in result as *“The quantitative measures of CAD and HF were not associated with all-cause mortality. (CAD, Z = 1.65, p = 0.10; HF, Z = 1.28, p = 0.20, sTable 3)”* (line 285)

#4. Results. Quality of studies should be more expanded and subgroup analysis for it should be added

Response:

Thanks for your comment. We had added subgroup analysis related to the mortality rates based on scores of Newcastle-Ottawa Scale Quality Assessment for included studies (NOS score =8 versus NOS score =9). (line 266-272) We revised the manuscript as following (line 266-272), *“ In respect of the impact of the quality of the included studies, analysis of the study with a NOS of eight showed a higher but non-significant increase in mortality risk in the DBB group compared to that in the NDBB group [Fig 3D, random effect, OR = 1.37 (95% CI, 0.93–2.0), p = 0.12]. In contrast, investigating the other three studies reporting a NOS score of nine demonstrated a non-significant reduction in mortality risk among those receiving DBBs compared with those taking NDBBs [Fig 3D, random effect, OR = 0.85 (95% CI, 0.71–1.02), p = 0.09].”*

**Reference**

1.	Tieu A, Velenosi TJ, Kucey AS, Weir MA, Urquhart BL. β-Blocker Dialyzability in Maintenance Hemodialysis Patients: A Randomized Clinical Trial. Clinical journal of the American Society of Nephrology : CJASN. 2018;13(4):604-11. Epub 2018/03/10. doi: 10.2215/cjn.07470717. PubMed PMID: 29519953; PubMed Central PMCID: PMCPMC5969458.

2.	Georgianos PI, Agarwal R. Pharmacotherapy of Hypertension in Chronic Dialysis Patients. Clinical journal of the American Society of Nephrology : CJASN. 2016;11(11):2062-75. Epub 2016/11/01. doi: 10.2215/cjn.00870116. PubMed PMID: 27797886; PubMed Central PMCID: PMCPMC5108184.

3.	Fravel MA, Bald E, Fraer M. Antihypertensive Agents in the Dialysis Patient. Curr Hypertens Rep. 2019;21(1):5. Epub 2019/01/20. doi: 10.1007/s11906-019-0909-z. PubMed PMID: 30659374.

4.	Velenosi TJ, Urquhart BL. Pharmacokinetic considerations in chronic kidney disease and patients requiring dialysis. Expert opinion on drug metabolism & toxicology. 2014;10(8):1131-43. Epub 2014/06/26. doi: 10.1517/17425255.2014.931371. PubMed PMID: 24961255.

5.	Shireman TI, Mahnken JD, Phadnis MA, Ellerbeck EF. Effectiveness comparison of cardio-selective to non-selective β-blockers and their association with mortality and morbidity in end-stage renal disease: a retrospective cohort study. BMC Cardiovasc Disord. 2016;16:60. Epub 2016/03/26. doi: 10.1186/s12872-016-0233-3. PubMed PMID: 27012911; PubMed Central PMCID: PMCPMC4807583.

6.	Assimon MM, Brookhart MA, Fine JP, Heiss G, Layton JB, Flythe JE. A Comparative Study of Carvedilol Versus Metoprolol Initiation and 1-Year Mortality Among Individuals Receiving Maintenance Hemodialysis. American journal of kidney diseases : the official journal of the National Kidney Foundation. 2018;72(3):337-48. Epub 2018/04/15. doi: 10.1053/j.ajkd.2018.02.350. PubMed PMID: 29653770; PubMed Central PMCID: PMCPMC6477681.

7.	Roux A, Aubert P, Guedon J, Flouvat B. Pharmacokinetics of acebutolol in patients with all grades of renal failure. European journal of clinical pharmacology. 1980;17(5):339-48. Epub 1980/05/01. doi: 10.1007/bf00558446. PubMed PMID: 7418713.

8.	Campese VM, Feinstein EI, Gura V, Mason WD, Massry SG. Pharmacokinetics of atenolol in patients treated with chronic hemodialysis or peritoneal dialysis. Journal of clinical pharmacology. 1985;25(5):393-5. Epub 1985/07/01. doi: 10.1002/j.1552-4604.1985.tb02864.x. PubMed PMID: 4031118.

9.	Flouvat B, Decourt S, Aubert P, Potaux L, Domart M, Goupil A, et al. Pharmacokinetics of atenolol in patients with terminal renal failure and influence of haemodialysis. British journal of clinical pharmacology. 1980;9(4):379-85. Epub 1980/04/01. doi: 10.1111/j.1365-2125.1980.tb01065.x. PubMed PMID: 7378254; PubMed Central PMCID: PMCPMC1429992.

10.	Kirch W, Köhler H, Mutschler E, Schäfer M. Pharmacokinetics of atenolol in relation to renal function. European journal of clinical pharmacology. 1981;19(1):65-71. Epub 1981/01/01. doi: 10.1007/bf00558387. PubMed PMID: 7461026.

11.	Seiler KU, Schuster KJ, Meyer GJ, Niedermayer W, Wassermann O. The pharmacokinetics of metoprolol and its metabolites in dialysis patients. Clinical pharmacokinetics. 1980;5(2):192-8. Epub 1980/03/01. doi: 10.2165/00003088-198005020-00006. PubMed PMID: 7363534.

12.	Niedermayer W, Seiler KU, Wassermann O. Pharmacokinetics of antihypertensive drugs (atenolol, metoprolol, propranolol and clonidine) and their metabolites during intermittent haemodialysis in humans. Proceedings of the European Dialysis and Transplant Association European Dialysis and Transplant Association. 1978;15:607-9. Epub 1978/01/01. PubMed PMID: 740698.

13.	Weir MA, Dixon SN, Fleet JL, Roberts MA, Hackam DG, Oliver MJ, et al. β-Blocker dialyzability and mortality in older patients receiving hemodialysis. Journal of the American Society of Nephrology : JASN. 2015;26(4):987-96. Epub 2014/11/02. doi: 10.1681/asn.2014040324. PubMed PMID: 25359874; PubMed Central PMCID: PMCPMC4378109.

14.	Wu PH, Lin YT, Kuo MC, Liu JS, Tsai YC, Chiu YW, et al. β-blocker dialyzability and the risk of mortality and cardiovascular events in patients undergoing hemodialysis. Nephrology, dialysis, transplantation : official publication of the European Dialysis and Transplant Association - European Renal Association. 2020;35(11):1959-65. Epub 2020/07/29. doi: 10.1093/ndt/gfaa058. PubMed PMID: 32719861.

15.	Bosco E, Hsueh L, McConeghy KW, Gravenstein S, Saade E. Major adverse cardiovascular event definitions used in observational analysis of administrative databases: a systematic review. BMC medical research methodology. 2021;21(1):241. Epub 2021/11/08. doi: 10.1186/s12874-021-01440-5. PubMed PMID: 34742250; PubMed Central PMCID: PMCPMC8571870.

16.	Hirata S, Kadowaki D. Appropriate drug dosing in patients receiving peritoneal dialysis. Contributions to nephrology. 2012;177:30-7. Epub 2012/05/23. doi: 10.1159/000336933. PubMed PMID: 22613912.
